# “A Different Ride”: A Qualitative Interview Study of Parents’ Experience with Early Diagnosis and Goals, Activity, Motor Enrichment (GAME) Intervention for Infants with Cerebral Palsy

**DOI:** 10.3390/jcm12020583

**Published:** 2023-01-11

**Authors:** Catherine Morgan, Nadia Badawi, Iona Novak

**Affiliations:** 1 Cerebral Palsy Alliance Research Institute, Specialty of Child and Adolescent Health, Sydney Medical School, Faculty of Medicine and Health, The University of Sydney, Sydney, NSW 2006, Australia; 2 Grace Centre for Newborn Care, The Children’s Hospital at Westmead, Sydney, NSW 2145, Australia; 3 Faculty of Medicine and Health, The University of Sydney, Sydney, NSW 2006, Australia

**Keywords:** cerebral palsy, qualitative interviews, parent experience, diagnosis, early intervention, GAME

## Abstract

Cerebral palsy is the most common physical disability of childhood, and early diagnosis followed by best practice early intervention is important for optimizing child and family outcomes. We investigated parents’ views of an early diagnosis of cerebral palsy (CP), followed by Goals, Activity, Motor Enrichment (GAME) intervention. Semi-structured interviews were conducted within a pilot randomised clinical trial. Transcriptions were analyzed using grounded theory. Participants were nine mothers whose infants had received GAME intervention because they were identified as being at high risk for cerebral palsy early in infancy. The parenting experience was described as a “different ride”. The diagnosis was devastating with many time-consuming challenges, but acceptance ensued. Parents wanted an early diagnosis, prognosis, and early intervention, despite the anxiety and workload, because it meant they could help. Parents perceived that GAME was beneficial because they were taught how to help; it was goal-based and home-based. They believed the collaboration and communication skills of the therapist shaped success. Future research should focus on a broader range of participants to understand parent’s experiences with key aspects of early intervention more fully.

## 1. Introduction

Cerebral palsy (CP) is the most common physical disability of childhood, with an incidence of 1.4/1000 live births in high-income countries [1]. CP is an umbrella term that covers a range of motor disorders resulting from an insult to the developing brain that can occur anytime during pregnancy through to early childhood [2]. Most often, the motor disorder of CP is accompanied by associated challenges including feeding, cognitive, and communication impairments. About half of all infants with CP are significantly unwell during the first weeks of life and require critical care in a neonatal intensive care unit (NICU). A significant proportion appear well at birth and are only identified when developmental delays are apparent. No matter what age, receipt of the diagnosis is a crisis event for parents, involving shock, anger, depression, and adjustment [3]. Chronic sorrow ensues, with some parents experiencing distress, while others experience relief [4]. In the last 5 years, the CP field has increasingly acknowledged the importance of an early diagnosis and early intervention. Three clinical practice guidelines now exist to guide: (1) making an early CP diagnosis [5] with 98% accuracy [6]; (2) provision of evidence-based CP-specific early interventions to harness neuroplasticity [7]; and (3) compassionate communication of difficult news to families [8].

In the past, a CP diagnosis was made late at 1–2 years of age [9]. Some clinicians were reticent to diagnose, apprehensive about false-positive diagnoses (reported as less than 5% [10]), and unsure about the effect of early diagnosis on parent–child bonding [9]. Late diagnosis meant some families experienced a stressful wait-and-see period between suspected CP and the start of intervention. There are numerous parent narratives about the lasting negative impact on families when the diagnosis was delivered sub-optimally with inadequate doctor–parent communication [11]. In contrast, there is also parental advice about how to do it well, informed by parental preferences [8]. It is now recommended that diagnostic conversations are wrapped up by developing a shared plan for early intervention, given that parents want to help and find delays between diagnosis and treatment unreasonable [8].

Parents of newly diagnosed infants often feel overwhelmed, and evidence indicates they are at a higher risk for chronic physical and psychological health problems, especially when caregiving demands are high [11]. Factors strongly related to parenting stress include high family needs; low family adaptability; and cognitive impairment of the child [12]. Caregiving burden stress can compound a pre-existing increased risk of attachment, responsivity, and psychological issues, common amongst mothers of infants born preterm [13], which accounts for 40% of all CP. It is known that infants at risk of disability, who have early exposure to a mother experiencing depression, have worse cognition and communication skills [14].

Despite the stress, parents of infants and young children diagnosed with CP describe feeling compelled to do everything they can to advance their child’s development [15]. Family-centred practice is the accepted standard of care when working with high-risk infants [16] and children with disabilities and recognises parental expertise and partners with parents in all aspects of intervention [17]. Early intervention strategies for infants at a high risk of disability should be incorporated into activities of daily living and involve the whole family unit to optimise learning and the generalisation of skills [18]. As well as supporting parents’ attachment to their baby, parental involvement is regarded as important to ensure that infants with developmental problems receive an adequate dose of intervention given that the home is the infant’s major learning environment [7,19]. Parents play a fundamental role in providing experiences that can enhance or undermine the mastery of skills and self-agency. A mother’s ability to match expectations and set up learning experiences at their child’s developmental level is known to influence the mastery of skills [20]. Clinicians vacillate between polarised views on whether involving parents in the delivery of intensive early interventions lowers or elevates stress. Newer approaches to early intervention for infants with CP involve both therapist-delivered and parent-delivered home-based intensive intervention using motor training [7]. Early evidence-based motor training is a strong recommendation in the clinical practice guidelines, given that all children with CP have a physical disability [7]. One such example is an intervention known as GAME, an acronym for goals, activities, and motor enrichment (protocol published elsewhere [21] and described in the methods). The establishment of collaboration appears protective. Parents who experience collaborative early intervention have less parenting stress, increased perceived competence, and more family-centred interactions compared to parents who did not experience collaboration [22].

With the shift to an accurate early diagnosis paired with good communication and immediate referral to early intervention, little is known about how parents’ experience these newer diagnostic and intervention practices. It is also crucial to establish that parent-delivered intensive early intervention does not cause harm to parents. We aimed to investigate the views of parents of infants who received an early diagnosis of CP followed by immediate intensive GAME intervention.

## 2. Materials and Methods

*Design:* This study was undertaken to develop a theoretical understanding of parent perspectives on early diagnosis and intervention within a pilot randomised controlled trial (RCT) of GAME [23]. A qualitative descriptive design with grounded theory was applied. Naturalism was the underpinning theoretical foundation—we aimed to stay true to the parent data but acknowledged our biases as researchers in the GAME RCT. We sought to provide a summary of participants’ views by conducting semi-structured qualitative interviews to collect the data, which were iteratively collected and constantly compared to identify and characterize common concepts about early diagnosis and intervention experiences and condensed into themes [24].

*Procedure:* Ethical approval was granted from the Human Research Ethics Committees of the University of Notre Dame Australia, Cerebral Palsy Alliance, and Sydney Children’s Hospital Network (Ethics No: 11CHW126). Full informed consent was obtained, and it was explained that non-participation or participation would not influence their infant’s ongoing care. Semi-structured interviews were held at the family home, at a time convenient for them, within 2 weeks of completing the GAME RCT primary end-point measures at 12 months of age. Other family members were sometimes present in the background but were not formally drawn into the interview. The lead author, a physiotherapist who had provided the GAME intervention, conducted the interviews.

*Participants:* We invited a consecutive series of parents of infants enrolled within the experimental arm of the GAME pilot to participate in semi-structured interviews about their experiences of early diagnosis followed by GAME intervention [23]. Infants enrolled within the GAME pilot were diagnosed with CP or “high-risk of CP” as identified by the General Movements Assessment (absence of fidgety movements at 9–18 weeks post term age) and/or neuroimaging indicative of likely permanent motor impairment as screened by a neurologist. Infants were between 3 and 6 months of corrected age at enrolment. Parents were invited to participate at the beginning of the infant’s participation in the GAME pilot.

*Data Collection:* The interviews were conducted between November 2013 and November 2014. The interview encouraged parents to describe their experiences and feelings, explain the aspects of the intervention that were important to them, and share their perspectives about receiving a CP or “high-risk of CP” diagnosis. As per the recommendations of Corbin [25], the interview guidelines were developed prior to data collection and are shown in Table 1. The questions were open-ended and were pilot tested with Participant One. New prompt questions were identified as a result of this interview and added to the subsequent interviews. The interviews were audio-taped and transcribed verbatim, with names removed to maintain anonymity.

*GAME Intervention*: The key features of GAME intervention include intensive motor training; coaching and education of parents; and assistance to modify the home environment to promote learning [21,23]. As per the protocol, parents were invited to identify motor or other developmental goals (e.g., babbling, playing with toys) that were important to them and were coached to assist their child to practice activities directed at goal attainment. The properties of objects and the built environment are exploited to positively evoke self-initiated repetitions of a desired motor behaviour. The collaboration also included supporting parents to be responsive to their child’s cues; problem solving for sleep and feeding issues that interfere with learning; and encouraging parents to ask questions about the current and future implications of their child’s diagnosis. GAME intervention was provided in the infant’s home at least fortnightly from enrolment (at 3–6 months of corrected age) until the child’s first birthday, by either or both the first and senior authors (physiotherapist and occupational therapist).

*Data Analysis:* To maximise rigour, analysis was conducted by two coders (CM, IN) following the staged approach proposed by Burnard [24]. First, transcripts, along with any field memos, were read through, and notes were made independently for immersion within the data. Next, transcripts were read again, with quotes, phrases, and words clustered into initial categories and broad headings assigned using open coding from the grounded theory approach. Then, categories related to each other were collapsed to reduce duplication and form themes. Agreement was established among the two coders on the direct quotes applying to the themes. Where the researchers disagreed on quotes, categories, or themes, the transcripts were reviewed and discussed using Burnard’s validity checks, with adjustments made until a consensus was reached.

## 3. Results

Of the 15 families in the experimental arm of the GAME pilot, 9 parents (all mothers) participated in the semi-structured interview study. Four families did not participate because they had relocated and thus withdrawn from the GAME pilot prior to the child’s first birthday; one family did not speak English, and one family was not pursued for interview as their infant was hospitalised at the time. Table 2 contains demographic information about both mothers and children who participated in the interviews. The parents’ occupations prior to the birth of the infant with CP varied, indicating socio-economic diversity. Interviews ranged in length from 10 to 52 min with a mean of 36.18 min per interview. Data saturation was reached by the ninth interview as no new relevant knowledge was being obtained.

### A Different Ride

Overall, the interview study findings revealed that parenting an infant with CP (or at high risk of CP) was a different parenting experience to the one that had been expected or previously experienced with other children: “It’s been a different ride”. Some parents alluded to experiencing stigma from social attitudes, making them concerned about leaving the house and wanting to avoid pity: “We weren’t afraid to know our son had cerebral palsy, but I’m not going to say I was proud.” Three major themes were identified. First, the journey from diagnosis to early intervention was emotional with mixed feelings. Second, parents perceived the importance of truth telling during diagnostic and prognostic information sharing. Third, parents described specific perceived benefits of GAME intervention.

## 4. Emotional Journey with Mixed Feelings

### 4.1. Devastating Diagnosis

Parents described the initial news of discovering that their baby was at high risk for cerebral palsy (CP) or confirmed to have CP as a traumatic and devastating experience. Most parents received this difficult news directly from the doctor as per the new guidelines. One parent uncovered the diagnosis indirectly from hospital reports. No matter how the news was communicated, parents described the diagnosis itself as “devastating”, “extremely overwhelming”, and “very hard” (Box 1).

Box 1 **Devastating diagnosis.**

- “It is traumatic, devastating news. It’s almost like telling a parent their child is dead. It is the same sort of grieving process because you’ve lost the child that you thought you were going to have. Even now, it is very hard for me to speak about it.”
- “I felt sad at being told he was high-risk for cerebral palsy. It was really upsetting, heart-wrenching, devastating, extremely overwhelming. I didn’t know what to do or how I would cope.”
- “It was devastating to find out. I went through a severe depression.”
- “It was shocking. And there was also the question, why me?”
- “I just got scared of that document. She had 14 out of 15 risk points or something like that.”

### 4.2. New Time-Consuming Challenges

The experience of parenting an infant with cerebral palsy was described as very different to previous parenting experiences for those who were not first-time parents. These experiences were related to their time in the Neonatal Intensive Care Unit (NICU); balancing the needs of other children; finding time to practice intervention strategies at home; and being intentional about promoting their child’s development. The experience was “more intense” and required: lots of appointments; being “more intentional” (e.g., structured developmental stimulation instead of spontaneous ontogenic learning as it was for siblings); “paying more attention”; and being “more technical”, with a tension between previous parenting practices and new recommendations. “*It took the fun out of it, that’s for sure*”. Parents talked extensively about the new and different time pressures (Box 2), plus, the challenges that came from learning what was needed and delivering on this. *“I had to push myself*” to meet these competing new responsibilities. Some felt “guilty” that they couldn’t fit everything into a day.

Box 2 **New time-consuming challenges.**

- “More time is the difference. It is more time-consuming and overwhelming for me.”
- “We are trying to still normalize our family and keep our other children’s routines. I have two other children, and my own partner.”
- “I think it is hard, for a parent to realize what they need to do for their child. It is very daunting. It does take a lot of your time.”
- “I still find it challenging every day to get things done. I also find it challenging to know if I only had this child, it would be so much easier to focus on him. I actually feel guilty. I need to do this; I need to do that. I need to get certain things done before I can get around to him.”
- “What I found the hardest was the fact that I have to find time, or I felt like I had to find time to do therapy every day.”

### 4.3. Shift to Acceptance

Over time, parents shifted towards acceptance. “*I slowly started to adjust to reality*”, even though parents knew “*It is not going to be a straight path*”. Acceptance of their child with a disability and their new unchartered family life came on different timelines depending on prior life experiences and beliefs about disability. Parents who had experienced chronic sorrow and loss during their fertility journey, such as previous miscarriages, death of a co-twin, or the decision to decline palliative care for their infant in the NICU appeared to reach acceptance at a faster rate. For these parents, the baby was a very wanted child irrespective of the cerebral palsy (Box 3), while other parents initially questioned whether they could develop acceptance for their child with a disability (Box 4).

Box 3 **Some parents shifted to acceptance early in their journey.**

- “I love that I chose this for my life.” [Mother who declined palliative care].
- “I decided to treat her has normal.”
- “Having a disability is not the end of the world.”
- “So we just make every effort we can to help him to develop, even he have this problem, he is still our son.” [Mother who lost twin at birth].

Box 4 **Other parents questioned whether they could reach acceptance.**

- “I didn’t know whether I would be fit to accept and embrace a child with disabilities.”
- “It was really hard in the beginning because I wanted it to be a different baby.”

## 5. Thankful for the Truth

All parents described experiencing a lot of waiting for news. “It was a waiting game”. Some parents suspected the diagnosis of cerebral palsy before it was given, and some managed the waiting by directly asking for a diagnosis (Box 5). Parents perceived that there was medical “hesitation” in giving the diagnosis, because it was especially difficult for doctors to give an accurate prognosis about the child’s future. “Nobody has the answers. No one can tell you.” Almost all parents observed that an uncertain prognosis was “frustrating”, “stressful”, and led to “anxiety” (Box 6). For one, the lack of news was hopeful.

Box 5 **Parents suspected the diagnosis, and some even asked for a diagnosis.**

- “When I did get the final diagnosis, it was no real surprise.”
- “The delay was obvious to me and scary.”
- “I had to ask for the diagnosis.”

Box 6 **Uncertainty brought anxiety.**

- “Not knowing what to expect, makes you think the worst.”
- “If you don’t know what future lies ahead, you worry more about their future. I was worried I wouldn’t know how to take care of him.”

Almost all parents concluded that having an early diagnosis was beneficial (Box 7). Parents wanted an early diagnosis despite the anxiety of prognostic uncertainty. Once a prognosis was available, the receipt of clear prognostic information brought about a reassuring turning point in their emotions (Box 8). An early diagnosis had emotional significance to parents because it meant parents knew they could help early, which was a universally valued notion (Box 9).

Box 7 **An early diagnosis was preferred.**

- “An early diagnosis is better, even if it is hard to accept.”
- “Early detection is always the best detection.”
- “I wanted the truth. I didn’t want negatives or lies. Knowing the truth is better than not knowing.”
- “It would have been worse later.”

Box 8 **Prognostic information helps manage parent emotions.**

- “A clearer prognosis decreases bad feelings.”
- “You don’t have certainty of outcome. I don’t want to have my hopes up.”

Box 9 **Early diagnosis meant early parent help.**

- “I preferred to know early, because then I can help as early as possible.”
- “I don’t regret finding out early, because it meant we could focus.”
- “It was a blessing he was diagnosed so early because he wouldn’t be where he is now.”

## 6. Perceived Benefits of GAME Intervention

Parents described the experience of GAME as “*a good kickstart*”. Many parents described feeling “lucky”, “thankful”, and positive about the intervention. Parents believed that GAME intervention “made a difference” and was “worthwhile”. Some parents described learning new ways of thinking about therapy and their role as a parent, for example: seeing play as therapy; or thinking about creating learning opportunities within daily tasks. “*It never leaves my mind*”. Many mothers described benefits to parents, including hope, reduced isolation, emotional support, and the fostering of acceptance and bonding (Box 10).

Box 10 **Benefits to Parents.**

- “It gave me hope. It made me more optimistic.”
- “Having someone come to the house made me feel less alone.”
- “It was a relief someone was helping.”
- “It helped me to accept his disabilities. To help with the abilities he does have and to improve his situation.”
- “We grew more attached. Because I sat with him, we bonded.”

### 6.1. Early Intervention Is a Must

Parents all agreed that children with cerebral palsy should have early intervention to maximize their chances, and that intervention should start as early as possible (Box 11). The process of engaging in early intervention reassured parents they are not alone. Parents reassured other parents “not to give up hope” and “hang in there” with the daily practice, because early intervention delivers “quick progress” that improves quality of life. They also recommend that parents seek support as well as learning to become an advocate for their child.

Box 11 **Early intervention is a must.**

- “It is a must.”
- “Do it.”
- “Go for it.”
- “Don’t think twice.”
- “Go hard.”

### 6.2. Hope from Progress

The initial shock and burden of the diagnosis was mitigated by the relief and hope in seeing their child make progress from early intervention. Parents felt that having early intervention from a trusted therapist who “knows” their child helped them learn to “watch”, “observe”, and “step back”, so as to identify improvements. “*I could see that she was progressing*” (Box 12). Once parents learnt to identify progress, they talked about the joy and importance of “celebrating achievements”, even the smallest of gains. Parents indicated that the identification of progress “gives focus” and motivated them to “strive harder” to practice tasks with their child. Where motor progress was slow, parents were able to identify other areas of progress and could even celebrate small gains (Box 13).

Box 12 **Progress brought relief, hope, and perseverance.**

- “Seeing improvements means hope and happiness. It promotes perseverance.”
- “Meeting milestones-this is what I hoped for.”
- “Feedback gives you comfort, direction and evidence. I found out he wasn’t going to be as severe as I worried.”

Box 13 **Learning to identify progress led to celebration.**

- “I needed to take a step back and watch. I didn’t believe it until I saw it.”
- “Him being able to sit is incredible.”
- “She has progressed a little bit. It is more fun with her that she’s interactive.”
- “I’ve noticed in play that she’s a little more skillful with her hand.”

### 6.3. Taught How to Help

All parents want to help. A key ingredient of the GAME protocol is to provide parent education and parent coaching in how to advance and enrich their child’s development. Over and above the benefits for the child receiving therapy, parents perceived that GAME constructively taught them how to help. “*We wanted to do what was best for her. It was her life. Being involved was really the only option*.” Parents described feeling unsure and unknowledgeable about “how to help” their child. They also described the emotional importance of being taught to realise they can and do help. “*You can actually help*!” and “*You feel like you have actually helped*.” Parents described learning how to set up for frequent and specific practice; how to adapt the environment; how to wait and not rescue their child too quickly so as to foster independence; how to build therapy into daily activities then “doing it together”; and how to read their child’s cues and emotions (Box 14). More specifically, parents also valued the environmental enrichment coaching that taught them how to select a toy for a learning moment to advance development. Parents described learning about the function of toys; matching a toy to a goal task; and using toys to advance or challenge their child’s practice. This led parents to organise their own toys differently, or buy new toys, or borrow toys from the toy library that matched the goals they chose for their child’s learning (Box 15).

Box 14 **Taught how to help.**

- “You gave us the guide about *how* to do the practice. You showed us how to practice standing up, which step, which foot first, which position. And *how* to do it is the most important thing.”
- “I wouldn’t have had the faintest idea what to do. You were teaching us both at the same time.”
- “I knew what he needed to do next.”
- “[I learnt] not rescuing (*ie helping straight away*) isn’t harmful. It allows learning and him to do it himself.”

Box 15 **Taught Toy Selection.**

- “[I learnt], using the bigger toys and the heavier toys when he’s sitting upright, tends to get him to sit up more and pay attention. Because he can’t pick up a big, heavy toy and put it in his mouth, he doesn’t lose balance as easily.” [Mother working on the goal of independent sitting].
- “I learnt [to choose] the right one.” [Mother matching a toy to a goal, rather than using toys solely for entertainment].
- “You showed me different sizes, different shapes, and different uses of toys.” [Mother working on the goal of grasp and grading the challenge].
- “I bought toys that encouraged both hands to be used rather than one.” [Mother working on bimanual hand function].

### 6.4. Goals Give Us the Drive

Parents mostly valued the goal-based approached to intervention. They perceived that it was beneficial to have tasks broken down, as it was less confusing and helped them to know what they were trying to achieve and focus upon. They observed that the process of defining goals drove them to practice the goals. “*The goals give us the drive*”. One parent disclosed that setting goals was emotionally confronting because it meant she was only asking for small things and thus only expecting small gains from her child.

### 6.5. Home Is Better

Parents perceived many benefits to GAME being provided in the home, including: the familiar environment gave the baby security and reduced stress; learning occurred at the optimal time for the baby’s wake cycle; parents felt more comfortable; it was more convenient and feasible for the mother (“*we wouldn’t have been able to leave the house as she hates the car*”); and the use of the baby’s own toys and home furniture meant it was customised to each family context and easier to incorporate and translate the therapy activities into real life (Box 16).

Box 16 **Home is Better.**

- “Home-visiting is really good thing for my baby. You know him well and choose the best time for him.”
- “It makes a lot more sense to have therapy in your own environment where you spend most of your time.”
- “I know how to incorporate therapy ideas using the home environment because that is the environment my child’s going to be in.”

### 6.6. Platform to Work Off

Parents believed the home program was “really helpful” advice and a “platform to work off”. They sensed the home program directly contributed to the child’s progress. “[Without the home program he] *never would have come out the way he is now.”* Notable features they liked were the actual photos of their child practicing, reinforcing the “how”; the notes as a “reminder to practice”; the regular program updates enabling them to grade the exercises; and the inclusion of siblings.

### 6.7. On the Same Page

Parents perceived important benefits from working together with the therapist as a collaborative team. We were “on the same page” with the “same person”. “It felt like it was your home too.” Parents experienced receiving “trusted advice” and “priceless knowledge and understanding” from therapists, which conferred hope. Parents described therapists as “teachers”, “mentors”, and “friends”. The therapist’s communication skills contributed to the collaboration and parent’s comfort. Parents valued sensing the therapist offered “genuine heartfelt care” for their baby and interpersonal safety, enabling parents to voice their opinions and say “*I can cry in front of you*”.

### 6.8. Therapy Isn’t Just about Therapy

Parents perceived that “*therapy isn’t just about therapy*”, but is also about the whole family’s needs and the therapist’s interpersonal skills. Parents advised therapists to: be supportive; be sensitive, as parents feel alienated and alone; be patient (with parents, the baby, and siblings) as “impatience makes us feel worse”; be “real, caring and respectful”; be cognizant of cultural social issues to maximize engagement; give regular feedback and encouragement to sustain engagement; relate to the child, not the diagnosis; and educate the family about what to realistically expect, as unrealistic expectations lead to pressure and bonding problems.

## 7. Discussion

This study provides information about parent’s experience with an early diagnosis followed by GAME intervention for their infant with cerebral palsy. Contrary to clinical assumptions [9], early diagnosis followed by GAME intervention did not interfere with bonding, but rather enhanced bonding. Nor did parental involvement in the delivery of early intervention have a harmful effect, but instead provided parents with guidance about how to help their child that was highly valued.

Our study indicates that cerebral palsy is a devastating diagnosis for parents to receive, regardless of the timing of diagnosis. Consistent with earlier research, the diagnosis is a “death” of a perceived child, resulting in chronic sorrow [4,26]. Diagnosis and involvement in early intervention reshapes the entire parenting experience, bringing about many additional and competing time pressures. This phenomenon has previously been termed “transformed parenting” [27]. Caregiving for a child with CP is known to have a significant effect on a family’s quality of life, including negative effects on social wellbeing, autonomy, finances, and marital wellbeing [28]. Despite this, parents universally agreed that they preferred an earlier diagnosis as it mobilised them to help earlier, which they believed was in their child’s best interest. Previous survey research has recommended an earlier diagnosis based on parental feedback [29], but little was known about the actual experience of an early diagnosis, given that earlier diagnosis is a newer practice. This is one of the first studies to confirm that they prefer an early diagnosis. Consistent with previous survey research, parents experienced frustration and anxiety when they sensed information was “hedged” to prevent prognostic inaccuracies [29]. In our study, parents expressed a desire to be told the truth early, not simply for information gathering, but so they could actively engage in helping their child. Moreover, parents experienced an emotional turning point when they received prognostic information and conversely found a lack of clarity about the severity of the disability, which led to more anxiety. Parents have previously expressed a desire for earlier and more prognostic information to help them with planning their family’s new future [30]. In the oncology field, honest conversations about prognosis are known to promote hope and support people to redefine their story [31]. The discovery of accurate early prognostic markers that give clinicians confidence plus the discipline of compassionate communication of prognostic information must continue to be a research focus for health professionals working in the cerebral palsy field.

With time, most parents resolve their reactions to the diagnosis [32]; however, new situations or transitions may trigger intensified sorrow symptoms [3]. All but one parent in our study had shifted towards acceptance of the disability before their infant was 12 months of age. This contrasts with previous research indicating that parents of younger children may still have unresolved reactions to the disability [32]. Parental satisfaction with the diagnostic process is, however, theorised to provide a portal through which families better adapt to the disability [33]. It is possible that for families in this pilot study, the combination of early diagnosis and GAME intervention cultivated resolution. Consistent with previous research, resolution with the diagnosis was strongly linked to parental feelings of attachment with their baby [34]. More research is therefore warranted exploring whether an early diagnosis paired with engagement in early intervention fosters acceptance.

The parents of our study identified core tenets of the Goals–Activities–Motor–Enrichment (GAME) approach that were beneficial to their child and to them as parents. Beginning with *goals*, parents valued the goal-based approach used within GAME intervention. Consistent with previous research, parents indicated that goals gave them “the drive” and motivation to practice at home [30]. Goal-based intervention is known to produce enjoyment and induce motivation, which in turn drives up the self-selected dose of practice [30], thereby harnessing neuroplasticity [35].

These parents emphasised the importance of being “taught how to help” via the *parent education and coaching* employed within the GAME approach. Parental self-efficacy and parenting competence are positively associated when parenting knowledge about development is high [36]. The coaching approach in GAME aims to foster efficacy, competence, and knowledge. These attributes are valued within GAME because when there is a mismatch between perceived high parent self-efficacy and low knowledge of child development, there is low responsivity to the infant results [36]. Parents were coached in *activity*-based practices targeting improvement in the goals parents selected. Activity-based interventions are known to produce large effect sizes in older children with cerebral palsy [37]. Parent’s perceived that GAME was producing progress, and this gave families hope. Hope, goals, and observation of progress are known to influence parents to practice more, which further increases the likelihood of improvements occurring [30].

Parents also valued the *environmental enrichment* aspects of the GAME approach. Parents appreciated that GAME was provided within the *home*. They sensed the home environment improved the baby’s performance, made the intervention more feasible for parents, and increased the likelihood of accurate practices leading to the generalisation of skills. These perceived benefits are consistent with previous home program research on older children with cerebral palsy, conducted using the same home program partnership model [30]. Home visiting is also known to be effective when the intervention directly targets issues of importance to both the parent and the child [38]. Parents in our study valued being taught toy selection, which was a process of showing them how to match the physical properties of a toy to their goal, the child’s ability level, and cues, so as to enhance their child’s developmental progress. This is not an unsurprising finding, given it is known that child development intervention effects are unlikely to occur unless mothers modify their interaction style [39]. Many aspects of the parenting interaction style are modifiable, but responsiveness to the child and their cues delivers the biggest gains for children [39]. In GAME, maternal responsivity is coached because high maternal responsivity is associated with elevated language, cognition, and social skills in children with disabilities [40].

The parents in our study emphasised the importance of the partnership and collaboration with the therapist to implement GAME within the home and into natural routines. Consistent with previous home program research, parents also identified that the therapist’s personal attributes and communication style were critical to success or failure [31]. Support from health professionals is known to enhance parents’ confidence [41]. Supportive and accepting behaviours from professionals “invigorate parenting”, especially when the sharing of mutual goals and tasks is also present [41]. Shared goals, tasks, and responsive parenting are core tenants of GAME. Conversely, the literature suggests that when parents do not experience these supports, there are consequences for the whole family, including difficulty in reaching acceptance for the child [41]. Prior clinician concern that parental involvement in early interventions such as GAME might harm bonding is not supported by the parents in this study. Parents of infants with CP are a vulnerable population, with mothers of children with CP experiencing higher rates of depression and anxiety. Anxiety is inversely correlated with the child’s sleep, and anxiety and depression are associated with lower parental self-efficacy [42]. Parents’ mental health is influenced by the child’s disability and workload, but also by the quality of support they receive [11]. It is therefore important for clinicians delivering early intervention to ensure that services target both the parent and the child’s needs in a collaborative and supportive mode. Additionally, it is important to provide an exemplar for accepting the child’s worth, as this goes beyond giving dignity to therapeutic transformation within the parent’s journey. These results suggest the therapist as a person is as important as the intervention itself.

*Limitations:* Given that this qualitative study was based on semi-structured interviews in a small number of participants, the data cannot be generalised to the wider CP population or indeed to children with other neuro-disabilities including various genetic disorders. In addition, the interviews were conducted by the lead author, who was also a treating clinician within the clinical trial, which may have influenced how parents responded during the interviews. Lastly, we did not interview families from the standard care arm of the trial, which is likely to have further enriched these data. In this pilot phase, we were interested to specifically explore parents’ experience with GAME intervention.

## 8. Conclusions

Parents want and prefer an early diagnosis of cerebral palsy, followed by early GAME intervention as it provides them with a conduit to help their child. Contrary to clinical assumptions, an early diagnosis promoted bonding. Furthermore, involvement with GAME early intervention was perceived as helpful and not harmful by parents. Parents recommended a collaborative, goal- and home-based intervention that included specific training on how to help their child.

## Figures and Tables

**Table 1:** Interview Guide.

Questions
What was it like getting the news about cerebral palsy?
Would you have preferred to wait a bit longer [for the diagnosis]?
Describe your experience of being involved with this project.
How did the therapy intervention you received help you as a parent interact with your child?
How did you know whether your child was making progress?
From your point of view what were the most important features of the intervention? (Prompt: How were these different for you and your child?)
What were the most important features of the partnership with the therapist(s) you worked with?
What would your advice be to other parents about to engage in early intervention for their baby?

**Table 2:** Characteristics of participant dyads.

Family	Maternal Age	Child Gender	Birth Order	Age of Confirmed Diagnosis	Cp Risk Factors	Type of CP	Type of Dwelling	Number of People in Home	Maternal Education
1	22 years	Male	2	12mths	Birth defects	monoplegia	Converted garage	4	High school
2	35 years	Female	1	10 mths	HIE	hemiplegia	townhouse	3	University
3	34 years	Male	3	8 mths	Extremely Preterm	hemiplegia	house	9	High school
4	35 years	Male	3	10 mths	Hydrocephalus	diplegia	house	5	High school
5	31 years	Male	1	12 mths	Preterm	triplegia	apartment	3	University
6	35 years	Male	9	8 mths	Preterm	quadriplegia	house	8	High school
7	39 years	Female	2	12 mths	Preterm	diplegia	apartment	4	University
8	30 years	Female	1	Not diagnosed	HIE	Mild neurological signs	apartment	4	University
9	35 years	Female	1	12 mths	asphyxia	quadriplegia	apartment	3	University

## Data Availability

Data sharing not applicable due to privacy and ethical restrictions.
